# An emerging research: the role of hepatocellular carcinoma-derived exosomal circRNAs in the immune microenvironment

**DOI:** 10.3389/fimmu.2023.1227150

**Published:** 2023-09-11

**Authors:** Huang-Zhen Xu, Xin-Yi Lin, Yun-Xian Xu, Hui-Bin Xue, Shu Lin, Tian-Wen Xu

**Affiliations:** ^1^ Department of Digestive Tumor, The Second Affiliated Hospital of Fujian Medical University, Quanzhou, China; ^2^ Centre of Neurological and Metabolic Research, The Second Affiliated Hospital of Fujian Medical University, Quanzhou, Fujian, China; ^3^ Group of Neuroendocrinology, Garvan Institute of Medical Research, Sydney, NSW, Australia

**Keywords:** hepatocellular carcinoma, exosome, circRNAs, microenvironment, immune escape

## Abstract

Hepatocellular carcinoma (HCC), the most common primary malignancy of the liver, is one of the leading causes of cancer-related death and is associated with a poor prognosis. The tumor microenvironment (TME) of HCC comprises immune, immunosuppressive, and interstitial cells with hypoxic, angiogenic, metabolic reprogramming, inflammatory, and immunosuppressive features. Exosomes are nanoscale extracellular vesicles that secrete biologically active signaling molecules such as deoxyribonucleic acid (DNA), messenger ribonucleic acid (mRNA), microribonucleic acid (miRNA), proteins, and lipids. These signaling molecules act as messengers in the tumor microenvironment, especially the tumor immunosuppressive microenvironment. Exosomal circRNAs reshape the tumor microenvironment by prompting hypoxic stress response, stimulating angiogenesis, contributing to metabolic reprogramming, facilitating inflammatory changes in the HCC cells and inducing tumor immunosuppression. The exosomes secreted by HCC cells carry circRNA into immune cells, which intervene in the activation of immune cells and promote the overexpression of immune checkpoints to regulate immune response, leading tumor cells to acquire immunosuppressive properties. Furthermore, immunosuppression is the final result of a combination of TME-related factors, including hypoxia, angiogenesis, metabolic reprogramming, and inflammation changes. In conclusion, exosomal circRNA accelerates the tumor progression by adjusting the phenotype of the tumor microenvironment and ultimately forming an immunosuppressive microenvironment. HCC-derived exosomal circRNA can affect HCC cell proliferation, invasion, metastasis, and induction of chemoresistance. Therefore, this review aimed to summarize the composition and function of these exosomes, the role that HCC-derived exosomal circRNAs play in microenvironment formation, and the interactions between exosomes and immune cells. This review outlines the role of exosomal circRNAs in the malignant phenotype of HCC and provides a preliminary exploration of the clinical utility of exosomal circRNAs.

## 1 Introduction

Hepatocellular carcinoma (HCC), the most common type of primary liver cancer, is characterized by the malignant, abnormal proliferation of hepatocytes. The development of HCC is typically associated with hepatitis B and C viral infections (1). Other causes of HCC include chronic alcohol consumption, smoking, diabetes, obesity, and exposure to aflatoxin B1 (AFB1) (2). HCC has an insidious onset with no specific signs or symptoms. It is usually diagnosed in patients presenting with severe jaundice and large-volume ascites; therefore, advanced HCC cannot be treated via conventional surgical resection, liver transplantation, or local percutaneous tumor ablation, resulting in high mortality rates (3).

The tumor microenvironment (TME) is the cellular environment of tumorigenesis, comprising a vast network of tumor cells, tumor-associated stromal cells (e.g., cancer associated fibroblasts (CAFs)), immune cells (e.g., T lymphocytes, B lymphocytes, tumor-associated macrophages (TAMs), dendritic cells (DCs), natural killer (NK) cells, neutrophils, and myeloid-derived suppressor cells (MDSCs)), and the cytokines and chemokines (CCLs) they secrete (4). The TME contributes to the development, progression, invasion, and metastasis of malignant tumors via interactions with tumor cells, and the immunosuppressive microenvironment plays an important role in the development of HCC (5). Under the guidance of HCC cells, various cytokines and CCLs in the TME recruit immune cells, which, in turn, play a role in inhibiting their killing function, promoting their apoptosis, and regulating their phenotype. The liver is an organ with a particularly large number of immune cells, including NK cells, gamma delta (γδ) T cells, and resident hepatic macrophages, among other cell types (6), all of which form a complex tumor immune-suppressive microenvironment, leading to immune escape and continuous proliferation of liver cancer cells. Exosomes may play important roles in these processes.

Exosomes are small nanovesicles with phospholipid bilayer membranes of approximately 50–150 nm in diameter (7). Exosomes contain a variety of biologically active substances, such as deoxyribonucleic acid (DNA), messenger RNA (mRNA), micro-ribonucleic acids (miRNAs), long non-coding ribonucleic acids (lncRNAs), circular ribonucleic acids (circRNAs), proteins, and lipids (7). HCC-derived exosomal circRNAs are extracellular vesicles (Evs) secreted by HCC cells that carry circRNAs. Recent studies report that tumor-derived exosomal circRNAs can act as important messengers and play a significant role in the formation of the tumor immunosuppressive microenvironment (8, 9). Exosomal circRNAs reshape the tumor microenvironment by prompting hypoxic stress response, stimulating angiogenesis, contributing to the metabolic reprogramming, facilitating inflammatory changes in tumor cells and inducing tumor immunosuppression (10, 11). Furthermore, immunosuppression is the final result of a combination of TME-related factors, including hypoxia, angiogenesis, metabolic reprogramming, and inflammation changes. In conclusion, exosomal circRNA accelerates the tumor progression by adjusting the phenotype of the tumor microenvironment and ultimately forming an immunosuppressive microenvironment (12). In addition, the HCC-derived exosomal circRNAs can affect HCC cell proliferation, invasion, and metastasis and the induction of chemoresistance by modulating the relevant HCC signaling pathways (10, 13). Thus, there is great value in studying their role in the carcinogenesis, development, diagnosis, and treatment of HCC.

This review aims to summarize the role of exosomal circRNAs in the TME of HCC, the process through which exosomal circRNAs interact with immune cells, and the role of exosomal circRNAs in promoting the malignant phenotype of HCC. This review could lead to novel strategies that involve targeting exosome pathways to improve the effects of immune system-related therapies in the treatment of tumors.

## 2 Exosome discovery and function

Exosomes are small vesicles with a double-membrane structure. They were first identified in mature sheep reticulocytes cultured *in vitro* and were named by the Australian scientist Johnstone (14). Exosomes are secreted by various cell types, including erythrocytes, macrophages, lymphocytes, fibroblasts, and tumor cells. In humans, exosomes are found in the blood, urine, saliva, breast milk, and almost all other bodily fluids (15). Exosomes mediate intercellular communication and form a complex, multicomponent communication network in the TME, playing important roles in regulating biological functions in the microenvironment (16).

### 2.1 Biological generation and release of exosomes

Exosomes are mainly produced via the endosomal pathway, beginning with early endosomes formed by plasma membrane invagination, including cell surface proteins and soluble proteins associated with the extracellular environment. In some cases, vesicles derived from the trans-Golginetwork (TGN) outgrowth can fuse with early endosomes (17, 18). In the following maturation process, early endosomes (Rab5 as a marker) mature to form late endosomes (Rab7 as a marker) through acidification and material exchange (19). The late endosomal membrane invaginates into the lumen and forms intraluminal vesicles (ILVs), resulting in multi-vesicular bodies (MVBs) with a characteristic multi-vesicular appearance. MVB can fuse with the plasma membrane and release ILVs into the extracellular environment (20); the released ILVs are called exosomes. It can also fuse with lysosomes/autophagosomes to degrade the MVB (21). The exosome biological generation consists of three steps: ILV generation, MVB transport, and exosome release. Furthermore, Rab, ESCRT (endosomal sorting complex required for transport, ESCRT), SNARE (soluble N-ethylmaleimide-sensitive fusion attachment protein receptor), and other endosomal transport and secretion-related proteins affect exosome generation (17, 19).

And then exosomes are subsequently internalized by recipient cells through receptor-mediated endocytosis, pinocytosis, phagocytosis, or fusion with the cell membrane, resulting in the direct release of their cargo into the cytoplasm and allowing for the transmission of molecular signals that alter the function and phenotype of recipient cells (22). Exosomes are novel transmitters of intercellular signaling, forming a complex multicomponent communication network in the TME (23). The functional status of immune cells in the TME is a key factor that affects their ability to induce an immune response and is a critical link in tumor development. In recent years, intensive studies on exosome functions have revealed that HCC-derived exosomes can reshape the TME through multiple mechanisms, enabling HCC cells to evade immune surveillance and ultimately promoting tumor proliferation and metastasis (24). As a component of the TME, exosomes can regulate the polarization and function of immune cells, transforming the immune response from a tumor suppressive to a pro-tumor phenotype (25, 26), ultimately promoting tumor proliferation and metastasis.

### 2.2 Biological functions of exosomal circRNAs

CircRNAs are endogenous non-coding RNAs found in all eukaryotic cells and are mainly produced via “disordered splicing” (also known as reverse splicing) of the precursor mRNAs (pre-mRNAs) (27). CircRNAs comprise a class of conserved closed loop structures without 5’ caps, 3’ multi-tails, or polyadenylated tails; this structure ensures that they are highly stable and are not easily degraded by nucleic acid exonucleases, resulting in tissue- and cell-specific properties (28). The main biological functions of circRNAs include; (a) competitive binding with miRNAs to regulate the expression of target genes. CircRNAs contain a large number of miRNA binding sites that can act as miRNA molecular sponges, preventing miRNAs from binding to mRNA targets and thereby up-regulating the expression of mRNA target genes (27). (b) CircRNAs can interact with RNA-binding proteins (RBPs) to regulate gene expression. Various RBPs are involved in RNA shearing, stability regulation, and mRNA translation. In the absence of circRNAs, RBPs bind to mRNA, leading to the translation of a large number of proteins. Because the molecular sequence of circRNAs contains specific RBP-binding sites, circRNAs bind to RBPs to form RNA–protein complexes, inhibiting the function of RBPs and the transcription of parental genes (27). (c) CircRNAs can regulate gene transcription or splicing. CircRNAs can bind to the U1 small nuclear ribonucleoprotein particle (U1 snRNP) via RNA–RNA interactions. More specifically, the U1 snRNP forms a complex with RNA polymerase II, allowing it to bind to transcription factor IIH; thus, by regulating the activity of RNA polymerase II, circRNAs can regulate transcription or splicing (29). (d) CircRNAs can regulate the translation of proteins. Although circRNAs are non-coding RNAs, some circRNAs can be translated by ribosomes to encode polypeptides with regulatory functions (28, 30).

(**Figure 1**).

**Figure 1:**
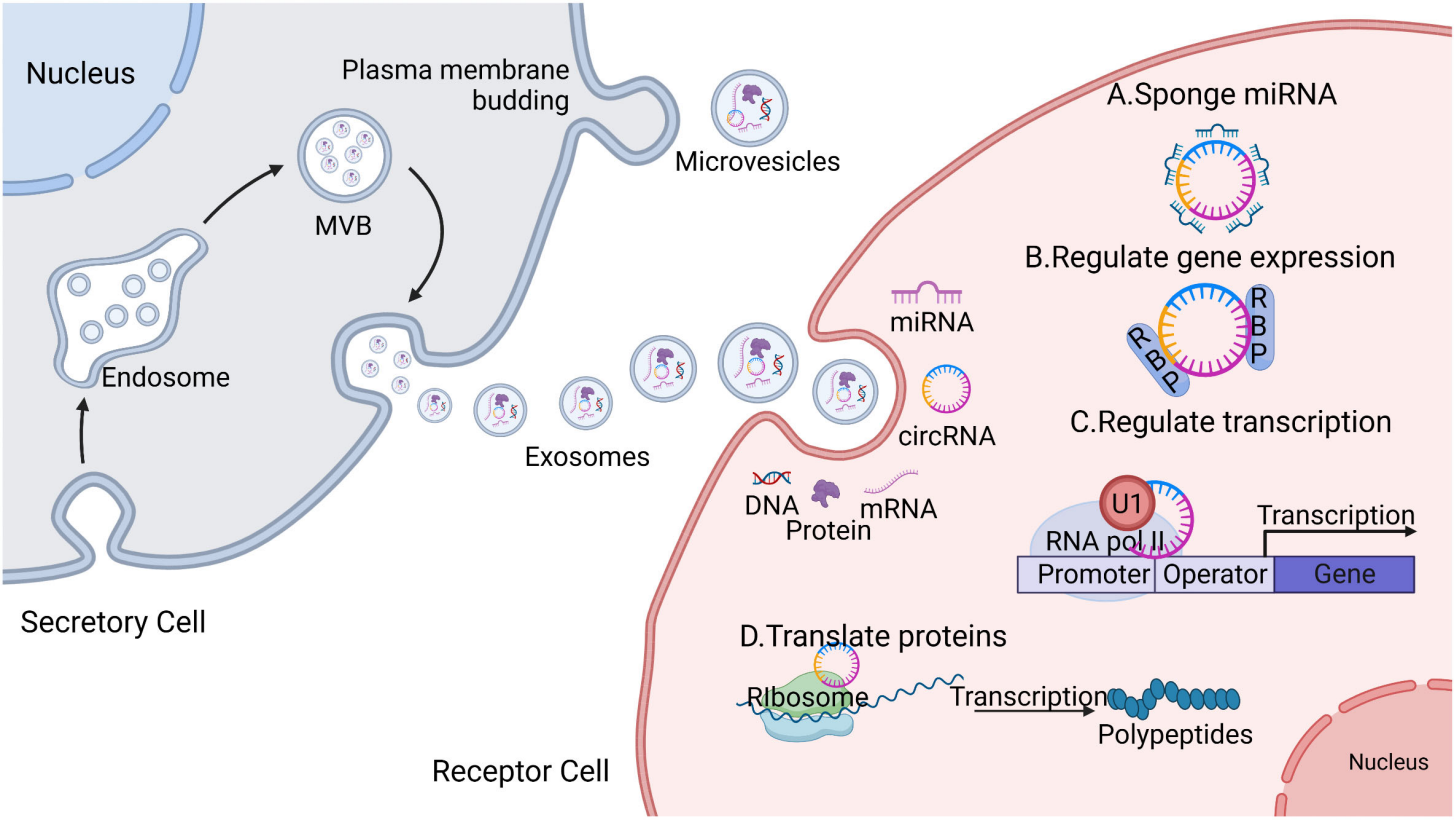
Secretory process and the biological function of circular ribonucleic acids (circRNAs). The secretory cells that bud inward to form an endosome gradually evolve into mature multi-vesicular bodies (MVBs), which fuse with the plasma membrane and release exosomes. Exosomes contain biologically active substances including but not limited to deoxyribonucleic acid DNA, messenger ribonucleic acid (mRNA), microribonucleic acid (miRNA), circRNA, and proteins, which are delivered to the recipient cells through endocytosis, allowing them to exert their biological effects. The main biological functions of circRNAs include the following: (A) acting as a competitive sponge for miRNA to regulate target gene expression; (B) interacting with RNA-binding proteins (RBPs) to regulate gene expression; (C) binding to the U1 small nuclear ribonucleoprotein particle (U1 snRNP), which forms a complex with RNA polymerase II to regulate gene transcription or splicing; and (D) some circRNAs can be translated by ribosomes into protein polypeptides and exercise regulatory functions.

Based on these biological functions, circRNAs play multiple roles in the biological processes of tumors, such as tumor cell proliferation, invasion, and metastasis, by promoting the formation of the TME and regulating the immunosuppressive microenvironment of HCC. Thus, circRNAs have the potential to become early diagnostic and therapeutic targets in various disease states (31). In summary, the biological function of carrier exosomes depends on the cargo they carry. Exosomal circRNAs play an important role in the immunosuppressive microenvironment of HCC because of their stability and differential expression, both of which affect the development of HCC.

## 3 HCC-derived exosomal circRNAs remodel the TME

**Figure 2:**
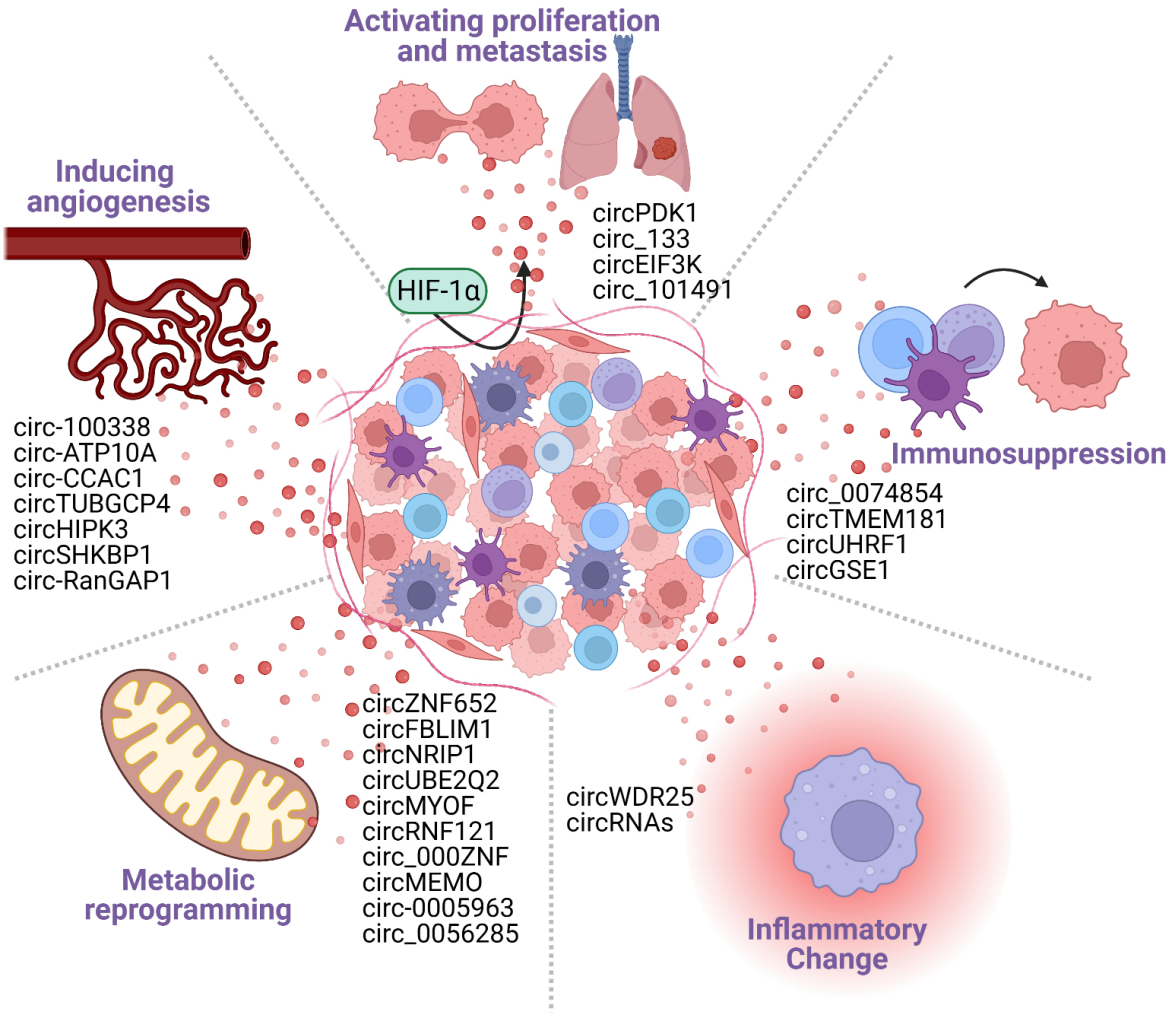
Cancer-derived exosomal circRNAs participate in the formation of the tumor microenvironment (TME). The TME includes tumor cells, tumor stromal cells, immune cells, and various cytokines secreted by them. The TME is characterized by the following five major features: hypoxia; angiogenesis; metabolic reprogramming; inflammatory changes; and immunosuppression. Tumor cell-derived circRNAs can promote the formation of TME features, generating an environment that is conducive to tumorigenesis and tumor development.

Tumor cell–immune cell interactions occur in the TME, characterized by its hypoxic, angiogenic, altered metabolism, inflammatory changes, and immunosuppressive conditions (32–35). Exosomal circRNAs have been reported to reshape the tumor microenvironment by prompting hypoxic stress response, stimulating angiogenesis (36), contributing to metabolic reprogramming (37, 38), facilitating inflammatory changes in the HCC cells (39) and inducing tumor immunosuppression. The exosomes secreted by HCC cells carry circRNA into immune cells, which intervene in the activation of immune cells and promote the overexpression of immune checkpoints to regulate immune response, leading tumor cells to acquire immunosuppressive properties (40). Furthermore, immunosuppression is the final result of a combination of TME-related factors, including hypoxia, angiogenesis, metabolic reprogramming, and inflammation changes. In conclusion, exosomal circRNA accelerates the tumor progression by adjusting the phenotype of the tumor microenvironment and ultimately forming an immunosuppressive microenvironment (**Figure 2**).

### 3.1 Exosomal circRNAs and hypoxia

Hypoxia is caused by the excessive proliferation of malignant cells and insufficient angiogenesis during tumor cell progression (41). And, hypoxia drives HCC progression and promotes the formation of an immunosuppressive microenvironment. Hypoxia inducible factors (HIFs) are the main regulators of an organism’s adaptive response to hypoxia (42). Under hypoxic conditions, HIFs recruit regulatory T lymphocytes (Tregs) and macrophages and promote sorafenib resistance (43). Besides, the HIF1α subunit also up-regulates programmed death-ligand 1 (PD-L1) expression in DCs (44), blocking the binding of DCs to CD28 on T cells, which inactivates them and facilitates immune escape in tumors (45). Furthermore, tumors recruit Tregs and type-2 conventional DCs (cDC2s) by increasing the expression of the chemokines C-X-C motif chemokine ligand 5 (CXCL5) and C-C motif chemokine ligand 20 (CCL20) in hypoxic environments (43, 46). The expression of CXCL5 has also been associated with recurrence, invasion, and overall survival (OS) in patients with HCC (47). The presence of sialoadhesin-positive (CD169^+^) macrophages is positively correlated with the number of Tregs (48). This particular type of macrophage recruits Tregs through the CC motif chemokine ligand 22–C-C motif chemokine receptor 4 (CCL22–CCR4) axis to induce immune tolerance, leading to tumor growth and metastasis (49). These findings suggest that hypoxia contributes to the formation of an immunosuppressive microenvironment in HCC.

Tumor cell-derived exosomal circRNAs are important players in hypoxia-induced non-coding RNA transcriptomics. Hypoxia-responsive circRNAs can transmit hypoxic signals from cancer cells to the surrounding stromal cells and the cells of distant organs to regulate hypoxic responses and promote hypoxic tumor progression and metastasis. For example, under hypoxic conditions, HIF1α can up-regulate the expression of phosphoinositide-dependent kinase-1 circRNA (circPDK1) in pancreatic cancer (PC). Exosomal circPDK1 acts as a scaffold to enhance the interaction between ubiquitin conjugating enzyme E20 (UBE2O) and bridging integrator 1 (BIN1), and it induces UBE2O-mediated degradation of BIN1, a protein that inhibits tumor progression by suppressing c-myc transcriptional activity. Through this mechanism, the presence of hypoxia-induced PC-derived exosomal circPDK1 can significantly promote PC cell proliferation, migration, and glycolysis (50). Similarly, hypoxia-induced exosomes containing circRNA 133 (circ-133) and circRNA eukaryotic translation initiation factor 3 subunit K (circEIF3K) can be transported to normoxic colorectal cancer (CRC) cells via the activation of the miRNA 133/Global H1/Ras homolog family member A (miR133a/Global-H1/RhoA) and miRNA 214/PD-L1 (miR-214/PD-L1) axes, respectively, to promote the proliferation and migration of CRC cells (51, 52). Hypoxia promotes high expression levels of circRNA 101491 (circ-101491) in the extracellular region of glioma cells, whereas activation of the circRNA 101491/miRNA 125b-5p/endothelin 1 (circ-101491/miR-125b-5p/EDN1) axis is associated with the malignant progression of glioma (53). There is a lack of studies on HCC-derived exosomal circRNAs and tumor hypoxia; however, based on the role of exosomal cellular communication, we speculate that HCC-derived exosomal circRNAs also play an important role in the formation of tumor-hypoxic environment. In summary, an increasing number of studies have shown that the specific hypoxic conditions of the TME can induce tumor cells to secrete specific exocrine circRNAs to promote the malignant phenotype of tumor cells. However, further investigations are required to determine whether hypoxia-responsive exosomal circRNAs can reverse the regulation of HIFs, promote the adaptation of tumor cells in response to hypoxic stress, and facilitate the formation of a tumor immunosuppressive microenvironment.

### 3.2 Exosomal circRNAs and angiogenesis

Rapid tumor growth is usually accompanied by abnormal proliferation of tumor neovascularization. Neovascularization provides large amounts of oxygen and nutrients, promoting tumor growth while also serving as an entry point through which metastatic cells can enter the systemic circulation, increasing the capacity for tumor invasion and metastasis. Vascular endothelial growth factor (VEGF) remains one of the most studied pro-angiogenic factors. Mechanistically, VEGF binds to its ligand (VEGFR) to activate a phosphorylation cascade that promotes endothelial cell proliferation and migration, leading to tumor neovascularization (54). Up-regulated expression of VEGF occurs in HCC and is positively correlated with tumor microvessel density, rapid disease progression, and a low survival rate (55, 56). In addition to promoting tumor angiogenesis, VEFG signaling also affects immune cells by inhibiting DCs (57) through the induced production of the tolerance enzyme indoleamine 2,3-dioxygenase (IDO) in DCs, preventing T cell infiltration into tumors, up-regulating programmed cell death protein 1 (PD-1) expression in CD8^+^ T cells, impeding the differentiation of T cells, and interfering with the function of cytotoxic T cells (CTLs) (56, 57).

Tumor-derived exosomal circRNAs can promote the formation of vascular endothelial cells and induce the formation of the neovascular lumen, thereby promoting the distant metastasis of tumor cells. Huang et al. (58) previously demonstrated that HCC-derived exosomal circ-100338 could be transferred to human umbilical vein endothelial cells (HUVECs), where it can bind its target receptor, promoting tumor neovascularization through the mammalian target of rapamycin (mTOR) signaling pathway. Circ-100338 expression positively correlated with the degree of tumor invasion, lung metastasis, and a poor patient prognosis. Yu et al. (59) used high throughput sequencing to screen for a clinically valuable exosomal circRNA of phospholipid-transporting adenosine triphosphatase 10A (circATP10A) in multiple myeloma (MM). The results revealed that the expression of this gene was significantly increased in patients with MM and was positively correlated with VEGF B (VEGFB) and marrow microvessel density (MVD) levels. Further analysis suggested that this could be achieved by targeting hsa-miR-6758-3p/hsa-miR-3977/hsa-miR-6804-3p/hsamiR-1266-3p/hsa-miR-3620-3p and regulating their downstream mRNAs, such as VEGFB, HIF1A, platelet-derived growth factor (PDGF), and fibroblast growth factor (FGF) to promote angiogenesis in MM. Similarly, Xu et al. (60) found elevated levels of the exosomal cholangiocarcinoma-associated circRNA 1 (circCCAC1) in bile and cholangiocarcinoma tissues. *In vivo* studies have demonstrated that the upregulation of Yin-Yang 1 (YY1) expression by the sponging of miR-514a-5p promoted progression and accelerated the tumorigenesis and metastasis of cholangiocarcinoma (CCA). Mechanistically, in a process closely related to neovascularization, the CCA-derived exosomal circCCAC1 undergoes translocation through the endothelial monolayer, disrupting the barrier’s integrity and inducing angiogenesis. Additional studies have shown that tumor-derived exosomal circRNAs are inextricably linked to tumor neovascularization. For example, CRC cells produce exosomal tubulin gamma complex-associated protein 4 circRNA (circTUBGCP4), which promotes angiogenesis and tumor metastasis by activating the protein kinase B (Akt) signaling pathway (61). Breast cancer (BRCA)-derived exosomal homeodomain-interacting protein kinase 3 circRNA (circHIPK3) enhances metadherin (MTDH) expression in endothelial cells by acting as a sponge for miR-124-3p, resulting in angiogenesis in BRCA cells (62). Similarly, Gastric cancer (GC)-derived exosomal short hairpin kinesin-binding protein circRNA (circshKBP) (63) and Ran guanosine triphosphatase-activating protein 1 circRNA (circ-RanGAP1) (64) have also been reported to act as specific miRNA sponges to promote neovascularization.

The above studies show that exosomal circRNAs are inextricably linked to tumor neovascularization. Tumor cell-derived exosomal circRNAs can accelerate the tumor metastatic process by promoting tumor angiogenesis. Thus, targeting exosomal circRNAs may be a promising strategy to reduce tumor metastasis and improve chemotherapy sensitivity.

### 3.3 Exosomal circRNAs and metabolism

Tumorigenesis causes a metabolic reprogramming of the microenvironment on which cells depend, including the activation of glycolysis, increased lipid metabolism, enhanced mitochondrial biosynthesis, and changes in other metabolic pathways (65). All of this reshapes the local TME and alters immune cells’ metabolic adaptation, leading to an immunosuppressive microenvironment (66, 67). Metabolite lactic acid and the acidic conditions of the TME reduce the low pH in NK cells, leading to pH dependent mitochondrial stress responses and metabolic dysfunction and the continuous accumulation of reactive oxygen species (ROS), leading to the apoptosis of NK cells (68). In addition, lactate is an important factor that regulates the biological behavior of T cells, inhibiting T helper (Th) cell function and reducing antitumor immune activity by preventing the binding of CXC motif chemokine receptor 3 (CXCR3) to its ligand (69). The monocarboxylate transporter (MCT) is a lactate transporter protein. Tregs can take up lactate from the TME via MCT and transfer it to the mitochondria, which participate in the tricarboxylic acid (TCA) cycle. This process can enhance both the expression of PD-1 and the immunosuppressive function of Tregs (70, 71). Cancer cells can evade CTL-mediated destruction by releasing lactate and kynurenine, thereby enhancing the immunosuppressive functions of Tregs and MDSCs (72). Lactate accumulation can also activate G-protein-coupled receptor 81 (GPR81) on endothelial cells, promoting angiogenesis and the immune escape of tumor cells (73). In conclusion, tumor metabolic reprogramming accelerates the immunosuppressive state of tumors, and exosomal circRNAs may mediate this process.

Metabolic reprogramming of HCC cells includes abnormally active glycolysis (also known as the Warburg effect), enhanced synthesis of fatty acids from scratch and weakened oxidation, and accelerated glutamine catabolism. These abnormal changes in metabolism provide HCC cells with intermediary substances and energy to meet their rapid growth, proliferation, and metastasis needs. Studies have shown that exosomal circRNAs are delivered between cancer cells to promote tumor metabolic reprogramming while forming a tumor microenvironment conducive to tumor growth. For example, the HCC exosomes zinc finger protein 652 circRNA (circZNF652) (37) and filamin-binding LIM protein 1 circRNA (circFBLIM1) (38) were found to activate glycolysis, promote proliferation, migration, and invasion of HCC cells, leading to a poor prognosis. Further, exosomal circRNAs have been shown to promote glucose metabolism not only in HCC but also in many other tumors. CRC-delivered exosomal nuclear receptor interacting protein 1 circRNA (circNRIP1) activates the AKT/mTOR pathway to promote the Warburg effect (74), whereas circ-0005963 promotes glycolysis and induces oxaliplatin resistance via the miRNA 122/pyruvate kinase muscle isozyme M2 (miR-122/PKM2) pathway (75). Similarly, GC-derived exosomal ubiquitin conjugating enzyme e2q2 circRNA (circUBE2Q2) promotes glycolysis through the miRNA 370-3p/signal transducer and activator of transcription 3 (miR-370-3p/STAT3) axis, altering the metabolic TME (76). Cancer-derived exosomes myoferlin circRNA (circMYOF) (77), circ-0056285 (78), really interesting new gene (RING) finger protein 121 circRNA (circRNF121) (79), circ_000ZNF (80), and mediator of cell mobility 1 circRNA (circMEMO1) (81) have also been found to be associated with the Warburg effect. In addition, alterations in the TME glycolytic pathway can drive the malignant phenotype of tumors by promoting the secretion of exosomal circRNAs by tumor cells. For instance, the Warburg effect promotes the release of exosomal circ-0072083 from drug-resistant glioma cells; mechanistically, this may promote temozolomide (TMZ) resistance by regulating miR-1252-5p-mediated glioma degradation and demethylation (82).

These results suggest that exosome-carrying circRNAs are useful for cellular communication between multiple cell types in TME. Besides glycolysis, tumor metabolic reprogramming also includes lipid and amino acid metabolism, and exosomes are involved in these metabolic processes (83, 84). However, the processes by which exosomal circRNAs of hepatocellular carcinoma secreted are involved in these metabolic reprogramming remain unclear and need to be further elucidated.

### 3.4 Exosomal circRNAs and inflammation

Inflammation is the driving force in HCC and represents one of the main features of TME. Multiple factors, such as hepatitis B and C viral infection and alcohol consumption, may lead to hepatic inflammatory changes and the development of HCC through the inflammatory liver lesion–liver fibrosis–hepatic cirrhosis pathway. Hepatic stellate cells (HSCs) are activated following hepatocyte damage, playing an important role in liver regeneration, inflammation, and fibrosis. Moreover, HSCs also play an important role in forming the immunosuppressive environment that promotes the development of HCC. HSCs help tumor cells evade immune surveillance by transforming macrophages and monocytes from the inflammatory (M1) to the immunosuppressive (M2) phenotype (85) and increasing the immunosuppressive populations of Tregs and MDSCs (86). Exosomes can act as a “bridge” between the inflammatory and immunosuppressive microenvironment of hepatocellular carcinoma; they promote each other to form a microenvironment conducive to tumor growth. Recent studies have shown that HSCs, which are important components of the TME, promote immune evasion through exosomal circRNAs and are associated with the proliferation and invasion of HCC (39). HSC-derived exosomal WD repeat domain 25 circRNA (circWDR25) were shown to increase the expression of cytotoxic T lymphocyte-associated protein 4 (CTLA-4) in HSCs and PD-L1 in HCC cells via the circWDR25/miR-4474-3p/arachidonic acid 15-lipoxygenase (ALOX15) and epithelial–mesenchymal transition (EMT) axis. PD-L1 produced in HCC cells binds to PD-1 on the surface of T cells to promote T cell apoptosis. Similarly, tumor-derived exosomes are involved in the formation of the tumor-inflammatory microenvironment and are closely associated with the immunosuppressive microenvironment. For example, HCC-derived exosomes can induce M2-type TAMs by activating the nuclear factor kappa-B (NF-κB) signaling pathway and inducing pro-inflammatory cytokine production. M2-type TAMs inhibit the expression of interferon-gamma (IFN-γ) and tumor necrosis alpha (TNF-α), two antitumor cytokines that exert inhibitory effects on tumor proliferation. Moreover, M2-type TAMs also exhibit a high degree of expression of the inhibitory receptors PD-1 and CTLA-4, further creating an inflammatory immunosuppressive microenvironment in HCC (87). It has also been demonstrated that tumor-derived circRNA can induce toll-like receptor 3 (TLR3), leading to stimulation of the NF-κB signaling pathway (88). However, further studies are required to identify the specific components of these exosomes. In summary, the effects of inflammation on HCC are multifaceted. We can utilize the relationship between exosomal circRNAs and the inflammatory microenvironment and immunosuppressive microenvironment to identify high-quality tumor markers for early prevention, detection, and treatment of HCC.

These studies have shown that immunosuppression is the final result of a combination of TME-related factors, including hypoxia, angiogenesis, metabolic reprogramming, and inflammation. Therefore, understanding the immunosuppressive processes in HCC could lead to identifying new targets or clinical therapies to treat such tumors.

## 4 Role of HCC-derived exosomal circRNAs on immune cells

**Figure 3:**
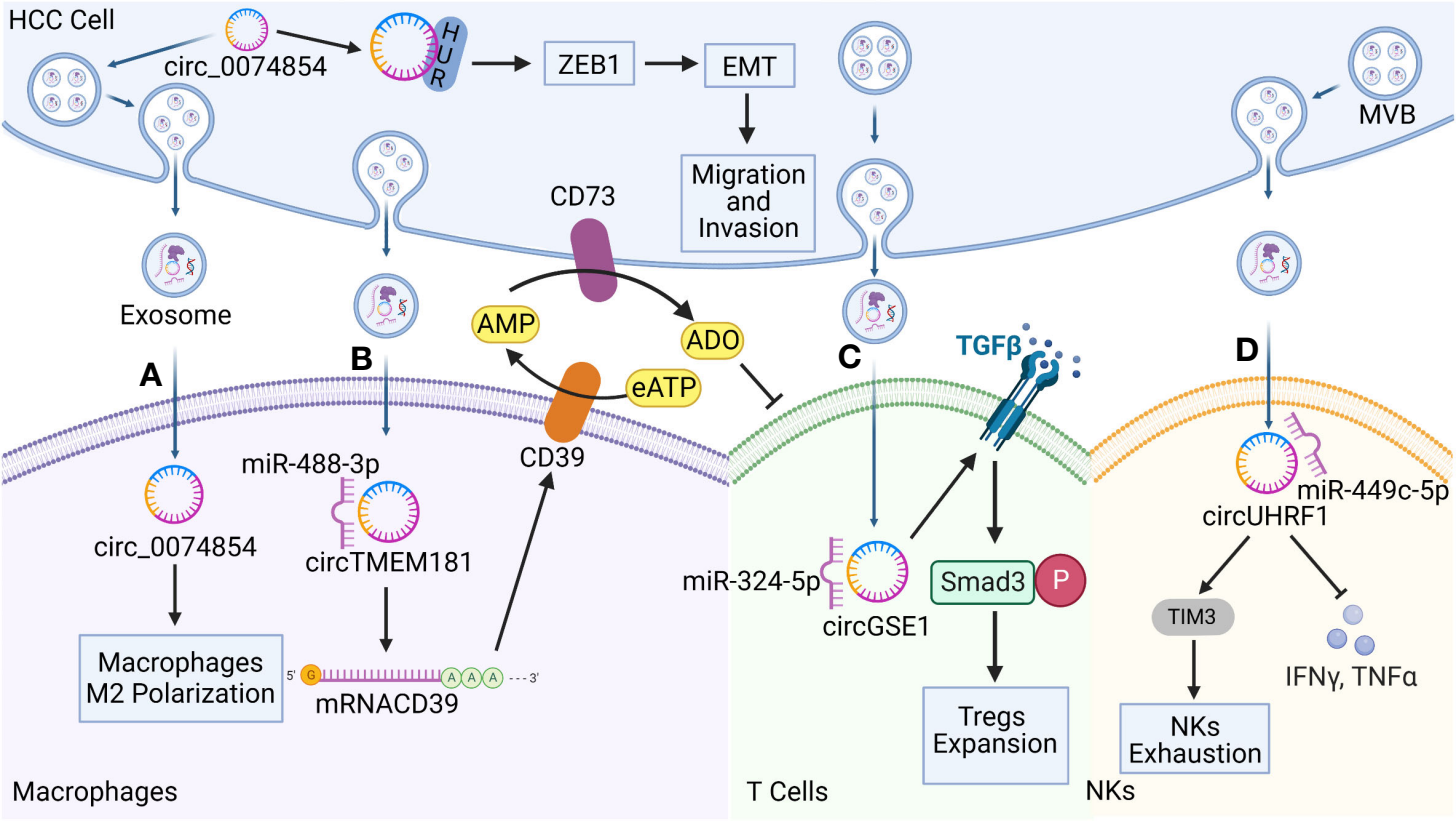
Functions of hepatocellular carcinoma (HCC)-derived exosomal circRNAs on immune cells in the TME. Exosomal circRNAs reprogram the biological behavior of various immune cells. **(A)** HCC-derived exosomal circ-0074854 was delivered to macrophages to induce pro-tumorigenic cell polarization toward the M2 phenotype. It also interacts with human antigen R (HuR) to promote HCC migration and invasion through the zinc finger E-box binding homeobox 1/epithelial–mesenchymal transition (ZEB1/EMT) pathway. **(B)** HCC-derived exosomal transmembrane protein 181 circRNA (circTMEM181) acts as a sponge for microRNA 488-3p (miR-488-3p) and up-regulates CD39 expression in macrophages, acting synergistically with CD73 to activate the extracellular adenosine triphosphate (eATP)–adenosine pathway in HCC cells, producing excessive quantities of adenosine that impair T cell function. **(C)** HCC derived exosomal Gse1 coiled coil protein circRNA (circGSE1) induces the amplification of regulatory T cells (Tregs) via the miR-324-5p/transforming growth factor beta receptor 1 (TGFBR1)/SMAD family member 3 (Smad3) axis. **(D)** HCC derived exosomal circUHRF1 is delivered to NKs, triggers NKs exhaustion via the miR-449c-5p/TIM3 pathway, and inhibits IFN-γ and TNF-α secretion.

Cytokines and chemokines of the TME interact with immune cells, causing HCC cells to evade immune surveillance and promoting HCC growth. Exosomes contain biologically active circRNAs that provide signals for intercellular communications. Thus, exosomal circRNAs are involved in regulating the microenvironment and exhibit immunosuppressive and tolerogenic properties, and the ability of tumors to evade immune surveillance is mediated through exosomes. The following section focuses on the immunosuppressive effects that exosomal circRNAs exert on immune cells (**Figure 3**).

### 4.1 Macrophages

Macrophages are important immune cells that are involved in the innate immune response. Under normal physiological conditions, macrophages are involved in phagocytosis and the digestion of cellular debris and pathogens, and they can activate other immune cells in response to pathogenic invasion. Macrophages are mainly categorized as M1 and M2 phenotypes. M2 macrophages are major components of tumor-infiltrating cells that suppress immune function, promote tumor cell growth and angiogenesis, and play key roles in the occurrence and development of HCC (89). Recent studies have shown that macrophages are important target cells of HCC-derived exosomal circRNAs. HCC cells deliver bioactive substances such as circRNAs to macrophages in the form of vesicles to allow them to evade immune surveillance. For example, Wang et al. (90) found that circ-0074854 could be transferred from HCC to macrophages via exosomes to stimulate the M2 polarization of macrophages. In addition, the stability of circ-0074854 is increased through interactions with human antigen R (HuR) (91), which, in turn, promotes EMT through the zinc finger E-box binding homeobox 1 (ZEB1) signaling pathway. EMT is a biological process through which cells lose their epithelial characteristics and transform into cells with an interstitial phenotype, thereby significantly enhancing their migration ability and mobility. EMT is also a key process in tumor metastasis. Circ-0074854 promotes the growth, migration, and invasion of HCC cells through both pathways synergistically. In addition, M2-type macrophages contribute to the production of an immunosuppressive TME and promote immune escape. The activation of adenosine signaling during metabolic reprogramming in the TME is an important immunosuppressive feature of HCC and is characterized by high levels of CD39 and CD73 expression (48, 92). CD39 and CD73 are two key enzymes that mediate the adenosine triphosphate–adenosine diphosphate (ATP-ADP) cycle, with CD39 degrading extracellular ATP (eATP) to ADP and AMP, and CD73 converting adenosine monophosphate (AMP) to ADO. As an end product of the CD39/CD73 metabolic pathway, adenosine is an important immunosuppressive factor that promotes the depletion of CD8^+^ T cells in the TME (93). The ATP–ADP cycle is promoted by circRNAs, which act as important signaling factors. Lu et al. (94) demonstrated that exosomal transmembrane protein 181 circRNA (circTMEM181) up-regulates the expression of CD39 in macrophages by acting as a sponge for miR-488-3p. They demonstrated that the expression of CD39 in macrophages was up-regulated, and HCC cells expressed CD73, resulting in the sequential activation of the eATP–adenosine pathway. Excessive accumulation of adenosine damages and depletes CD8^+^ T cells, resulting in resistance to PD-1 antibodies. Meanwhile, exosomal circTMEM181 expression has been found to be associated with a poor prognosis in patients with HCC and can be used as an independent prognostic indicator of HCC.

### 4.2 NK cells

NK cells play a key role in a host’s innate and adaptive immune defenses against tumors through their cytotoxic effects. NK cells can eliminate tumor cells by inducing apoptosis through perforin or granzymes, death receptors, antibody-mediated cytotoxicity, and cytokine production (95). Furthermore, the density and activity of NK cells in the TME is correlated with patient prognosis in various cancers (96), and HCC derived exosomal circRNAs have been reported to target NKs to promote the formation of an immunosuppressive microenvironment and drive the development of malignant tumor phenotypes. Zhang et al. (97) found that exosomal ubiquitin-like with PHD and RING finger domains 1 circRNA (circUHRF1) was predominantly secreted by HCC cells, with high levels of exosomal circUHRF1 being associated with reductions in the proportion of NK cells and the tumor infiltration rate of NK cells. HCC-derived exosomal circUHRF1 not only induces NK cell apoptosis by degrading miR-449c-5p to up-regulate T cell immunoglobulin and mucin domain-containing protein 3 (TIM-3) expression, but it also inhibits IFN-γ and TNF-α secretion by NK cells. TIM-3 is an important immune checkpoint molecule that suppresses antitumor immunity, and it also serves as a major marker of immune cell dysfunction (98). CircUHRF1 promotes the dysfunction and induces the apoptosis of NK cells, and drives immunosuppressive activity in HCC, all of which are closely associated with a poor prognosis. Furthermore, circUHRF1 may contribute to the resistance to anti-PD-1 immunotherapy in patients with HCC.

### 4.3 T cells

T cells are important players in the tumor immune response and are mainly categorized as CD4^+^ and CD8^+^ T cells. CD4^+^ T cells produce and release antibodies and participate in the antitumor effects mediated by CD8^+^ T cells. However, CD4^+^ T cells differentiate into Tregs, triggering the corresponding conditions that facilitate immune escape behavior. Forkhead box P3 (Foxp3) is a key regulator of the development and function of Tregs, leading to the inhibited expression of granzyme B in CD8^+^ T cells, thereby impairing the ability of CD8^+^ T cells and CTLs to destroy tumor cells (99). Recent studies have shown that exosomal circRNAs from HCC cells are involved in this pathway. Mechanistically, HCC cells secrete exosomal Gse1 coiled coil protein circRNA (circGSE1), which creates an immunosuppressive microenvironment by inducing the expansion of Tregs. HCC cells secrete exosomal circGSE1, which acts as a sponge for miR-324-5p and, in turn, activates the TGFß receptor 1 (TGFBR1)/SMAD family member 3 (Smad3) signaling pathway. Smad3 acts as an enhancer to induce the expression of FOXP3, which promotes the expansion of Tregs. In contrast, HCC-derived exosomal circGSE1 was shown to promote HCC progression, indicating that cicrGSE1 could represent a potential target for HCC immunotherapy (100). In addition to their role in the communication network that helps HCC cells evade immune surveillance, exosomal circRNAs commonly play similar roles in other tumor types. For example, exosomal circ-0069313 promotes its function by maintaining the level of Foxp3 transferred to Tregs in oral squamous cell carcinoma, and Foxp3 degradation can be inhibited in Tregs via the sponging of miR-325-3p; both of these mechanisms contribute to immune evasion by tumor cells (101).

In summary, exosomal circRNAs can transmit information between tumor and immune cells via exosomal secretion. By interfering with the activation of immune cells and the expression of immune checkpoints that regulate the immune response, exosomal circRNAs help tumor cells acquire immunosuppressive properties and indirectly promote the proliferation, invasion, metastasis, and progression of tumor cells. Therefore, exosomal circRNAs have the potential to be an effective method to improve the sensitivity of immunotherapy.

## 5 The role of exosomal circRNAs in the development of HCC

**Figure 4:**
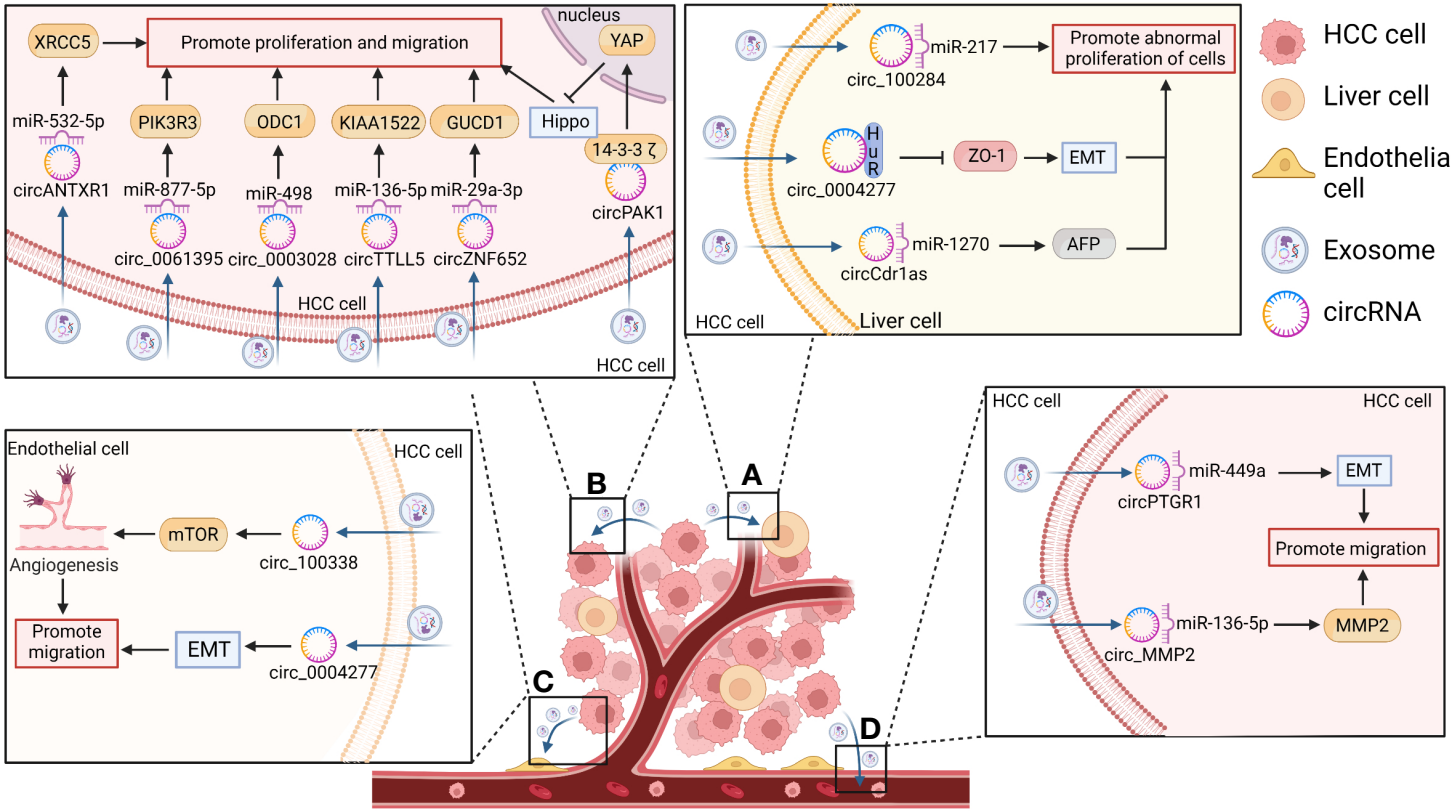
The function of exosomal circRNAs in HCC. CircRNAs secreted by HCC cells act on the corresponding recipient where they exert play biological effects related to the promotion of hepatocarcinogenesis and the proliferation, invasion, and metastasis of HCC. **(A)** CircRNAs enter the surrounding normal cells and promote the abnormal proliferation of liver cells and hepatocarcinogenesis by inducing epithelial– mesenchymal transition (EMT) (e.g., in the case of circ-0004277) or by increasing alpha fetoprotein (AFP) expression [e.g., in the case of cerebellar degeneration associated protein 1 antisense transcript circRNA (circ-Cdr1as)]. **(B)** CircRNAs act on themselves in turn to promote HCC proliferation, invasion, and metastasis by acting as sponges for their corresponding miRNAs [e.g., Anthrax toxin receptor 1 circRNA (circANTXR1), circ-0061395, circ-0003028, tubulin tyrosine ligase like 5 circRNA (circTTLL5), and zinc finger protein 652 circRNA (circZNF652)] or by blocking the Hippo/Yes-associated protein 1 (YAP) pathway (e.g., p21-activated kinase 1 circRNA (circPAK1)). **(C)** CircRNAs can also enter vascular endothelial cells in the form of exosomes to promote HCC metastasis via the EMT pathway (e.g., circ-0004277) or by increasing tumor neovascularization through the mammalian target of rapamycin (mTOR) pathway (e.g., circ-100308). **(D)** CircRNAs can also promote the metastatic ability of HCC cells [e.g., prostaglandin reductase 1 circRNA (circPTGR1) and matrix metallopeptidase 2 circRNA (circMMP2)].

The studies summarized in this review suggest that exosomal circRNAs are involved in the formation of TMEs. Furthermore, by regulating immunosuppression and immunosurveillance mechanisms, exosomal circRNAs are associated with increased tumor recurrence and enhanced tumor cell survival (13). The means through which they can regulate the development of HCC and affect tumor proliferation, invasion, metastasis, and chemotherapeutic drug resistance will be explored further in this section. (**Table 1** and **Figure 4**).

**Table 1:** The function of exosomal circRNAs in HCC.

Exosome component	Pathway/mediator	Function	References
circZNF652	miR-29a-3p/GUCD1	Promote glycolysis, proliferation, invasion, and migration	(37)
circFBLIM1	miR-338/LRP6	Promote glycolysis, proliferation, invasion, and migration	(38)
Not mentioned	NF-kB	Promote M2 TAMs polarization	(87)
circ-0074854	HuR/ZEB1/EMT	Promote M2 TAMs polarization, proliferation, invasion, and migration	(90)
circWDR25	miR-4474-3p/ALOX15 and EMT	Induce T cells apoptosis, proliferation, and invasion	(39)
circTMEM181	ATP-adenosine pathway	Promote T cells dysfunction and PD-1 resistance	(94)
circGSE1	miR-324-5p/TGFBR1/smad3	Promote Tregs, proliferation, invasion, and migration	(100)
circUHRF1	miR-449c-5p/TIM-3	Induce NKs apoptosis and PD-1 resistance	(97)
circ-100338	mTOR	Promote angiogenesis, invasion, and metastasis	(58)
circPAK1	Hippo/YAP	Promote angiogenesis, proliferation, invasion, migration and lenvatinib resistance	(102)
circ-100284	miR-217	Promote proliferation	(103)
circ-MMP2	miR-136-5/MMP2	Promote migration	(104)
circ-0004277	Inhibit ZO-1 and promote EMT	Promote proliferation and migration	(105)
circANTXR1	miR-532-5p/XRCC5	Promote proliferation and migration	(106)
circTTLL5	miR-136-5p/KIAA1522	Promote proliferation and migration	(107)
circCDR1AS	miR-1270/AFP	Promote proliferation and migration	(108)
circPTGR1	miR-449a/MET	Promote invasion and migration	(109)
circZFR	STAT3/NF-κB	Promote proliferation and DDP resistance	(110)
circ-0061395	miR-877-5p/PIK3R3	Inhibit cycle and apoptosis, promote proliferation and migration	(111)
circ-0003028	miR-498/ornithine decarboxylase 1	Inhibit apoptosis, promote proliferation and migration	(112)
circ-0051443	miR-331-3p/BAK1	Inhibit the malignant biological behavior of HCC	(113)
circSORE	YBX1/PRP19	Sorafenib resistance	(114)

### 5.1 Regulation of the proliferation of HCC cells

Dysregulation of proliferation is an important factor in tumor cell transformation. Therefore, an increasing number of studies have investigated the mechanisms through which the cell cycle can be regulated, and the role of exosomal circRNAs in the regulation of tumor cell proliferation has been slowly revealed in recent years (13, 29, 115). Human liver epithelial cells (L-02) undergo malignant transformation after longterm exposure to arsenite, resulting in the overexpression of circ-100284. Thus, circ100284 may be transferred from L-02 cells to normal cells via exosomes. In normal hepatocytes, exosomal circ-100284 accelerates cell cycle progression and promotes cell proliferation by interacting with miR-217, implying that exosomal circ-100284 plays a messenger role in intercellular communication and suggests an arsenite-induced oncogenic mechanism (103). Furthermore, Zhu et al. (105) demonstrated that circ0004277 was significantly up-regulated in HCC cells, tissues, and plasma exosomes compared to its levels of expression in normal hepatocytes. HCC-derived circ-0004277 was able to reach the surrounding normal cells through exosomal cell communication. It competitively binds to HuR, thereby blocking the translation of zona occludens 1 (ZO-1) and promoting EMT to facilitate the migration of HCC cells. ZO-1 is a tight junction protein that serves as an essential structure for maintaining the mechanical barrier and permeability of the mucosal epithelium, and low expression levels of this protein facilitate the metastasis of tumor cells.

Similarly, HCC-derived exosomal Anthrax toxin receptor 1 circ RNA (circANTXR1) promotes HCC proliferation and metastasis by up-regulating X-ray repair cross-complementing 5 (XRCC5) by acting as a sponge for miR-532-5p (106). Circ-0061395 regulates the expression of phosphoinositide-3-kinase regulatory subunit 3 (PIK3R3) through competitive binding to miR-877-5p. Through this mechanism, HCC-derived exosomal circ-0061395 can inhibit HCC cell cycle progression and apoptosis, promoting the proliferation, invasion, and migration of HCC cells (111). Other recent studies have shown that HCC-derived exosomal circ-0003028 inhibits apoptosis and promotes the proliferation and metastasis of HCC cells by regulating the miR-498/ornithine decarboxylase 1 (ODC1) axis (112). HCC-derived exosomal tubulin tyrosine ligase like 5 circRNA (circTTLL5) promotes the proliferation and metastasis of HCC cells through the miR-136-5p/KIAA1522 axis and promotes tumor growth in mice (107). HCC-derived exosomal circZNF652 promotes proliferation, migration, and invasion of HCC cells via the miR-29a-3p/GUCD1 axis (37). However, exosomal circ0051443 has been reported to inhibit malignant biological behavior through exosomal delivery from normal cells to HCC cells, where it competes with miR-331-3p for binding, up-regulates BAK1 expression, promotes apoptosis, and disrupts the cell cycle. Therefore, exosomal circ-0051443 could serve as a possible role as a predictor and potential therapeutic target for HCC (113).

The above studies show that exosomal circRNA is involved in intercellular communication during the proliferation and apoptosis of tumor cells and helps promote tumor development. If the traditional serum diagnostic markers of HCC, AFP, and exosomes are jointly applied to the diagnosis of early-stage HCC, the diagnostic rate of early-stage HCC patients can be improved, which is crucial for prolonging the survival time of HCC patients.

### 5.2 Regulation of HCC invasion and migration

The unique intercellular communication mechanisms of exosomes, which are important mediators of message transfer between HCC and other cell types, may generate a favorable microenvironment for HCC invasion and metastasis (13, 29, 115). The aforementioned HCC-derived ectodomain circRNA-100338 was found to promote angiogenesis in HCC. In addition, the results of a cross-pore invasion assay performed by Huang et al. (58) confirmed that exosomal circRNA-100338 could promote the invasion of HCC cells, and its expression levels were positively correlated with the occurrence of lung metastases following radical hepatectomy. These results suggest that exosomal circRNA-100338 enhances HCC cell invasion and metastasis by promoting angiogenesis. Yang et al. (108) also showed that cerebellar degeneration-associated protein 1 antisense transcript circRNA (circCdr1as) was highly expressed in HCC cells and tissues. Exosomes containing circCdr1as were also capable of transmitting and delivering information from HCC cells to surrounding normal cells and could promote their proliferation and migration by promoting the expression of alpha-fetoprotein (AFP) by acting as a sponge for miR-1270. Recently, a new circRNA was identified, dubbed p21-activated kinase 1 circRNA (circPAK1); the study showed that it could promote HCC cell proliferation, migration, invasion, and angiogenesis by inactivating the Hippo signaling pathway (102). Exosomal circPAK1 from HCC cells competes for binding sites between 14-3-3ζ protein and YAP, which may impair the recruitment and cytoplasmic fixation of 14-3-3ζ to YAP and promote the cytoplasmic localization of YAP, which is a switch protein that plays a central role in the Hippo signaling pathway. The Hippo–YAP signaling pathway is a newly discovered growth/regulatory signaling pathway that inhibits cell growth, proliferation, and apoptosis. Abnormal signaling in this pathway facilitates tumorigenesis. Wang et al. (109) demonstrated that the upregulated expression of exosomal prostaglandin reductase 1 circRNA (circPTGR1) via secretion of cells of the highly metastatic HCC cell line LM3 was associated with a poor patient prognosis. When LM3 cells were co-cultured with less metastatic HepG2 and 97L HCC cells, the less metastatic or non-metastatic cells exhibited a greater ability to invade and migrate. In addition, HCC-derived exosomal matrix metallopeptidase 2 circRNA (circMMP2) reportedly promotes HCC metastasis by up-regulating MMP2 via the sponging of miR-136-5p (104).

The studies above suggest that high expression of exosomal circRNA is associated with tumorigenesis and is closely related to the poor prognosis of HCC. Therefore, dynamic detection of exosomal circRNA levels in postoperative HCC patients can effectively reduce the risk of recurrence and improve prognosis.

### 5.3 Regulation of drug resistance in HCC

The resistance of tumor cells to therapeutic agents is the main reason for chemotherapeutic failure in patients with HCC, especially in those with advanced HCC. Sorafenib, Adriamycin, and platinum are conventional systemic or local chemotherapeutic agents for the treatment of HCC, although there is a high degree of resistance to them. Exosomal circRNAs, as important components of intercellular information exchange, can interact with various tumor microenvironment cells and act as miRNA sponges or protein scaffolds, among others, to regulate cancer drug resistance and influence tumor progression (116, 117). Recently, it was reported that exosomes can transfer circRNAs from drug-resistant cells to sensitive cells and participate in regulating cancer drug resistance by multiple mechanisms. In sorafenib resistant HCC cells, circSORE promotes sorafenib resistance via exosomes in HCC cells (114). Mechanistically, sorafenib-resistant circRNA from HCC cells (circSORE) binds to Y box binding protein 1 (YBX1), a master oncogenic protein present in the cytoplasm, blocking the nuclear interaction between YBX1 and the E3 ubiquitin ligase pre-RNA processing factor 19 (PRP19), thereby preventing the PRP19-mediated degradation of YBX1. Additionally, in different mouse models of HCC that involved circSORE silencing by small interfering RNA (siRNA) injection, the sorafenib resistance was significantly overcome, providing a potential strategy to combat sorafenib resistance clinically. The aforementioned HCC-derived exosomal circPAK1 was also found to induce lenvatinib resistance via its translocation through exosomal secretion from lenvatinib-resistant cells to sensitive cells (102). Mouse models and various pharmacological approaches also suggest that the HCC-derived exosomal circUHRF1 (97) and circTMEM181 (94) mediate the resistance to anti-PD-1 treatment in HCC. Some studies have reported that zinc finger RNA binding protein circRNA (circZFR) is secreted by CAFs; upon delivery to HCC cells, it promotes hepatocyte growth and enhances cisplatin (DDP) resistance via the STAT3/NF-κB pathway (110). All of these studies suggest that exosomal circRNAs play an important role in driving chemoresistance in HCC, and they may lead to novel strategies to improve chemotherapeutic outcomes in HCC. The studies above indicate that exosomal circRNAs induce drug resistance in HCC cells. Therefore, these identified exosomal circRNAs may be potential therapeutic targets for overcoming drug resistance. By affecting the synthesis and secretion of exosomes and their uptake by target cells, the drug resistance of tumors can be ameliorated, and the clinical benefits to patients can be enhanced.

Additional studies indicate that exosomal circRNAs play a larger role in the induction of chemotherapeutic resistance. For example, NSCLC-derived exosomal vacuole membrane protein 1 circRNA (circVMP1) (118), carboxypeptidase A4 circRNA (circCPA4) (119), phosphatidylinositol-4-phosphate 5-kinase type 1A circRNA (circPIP5K1A) (120), circ-0014235 (121), and circ-0008928 (80) were shown to be delivered to sensitive cell lines to promote DDP resistance. Exosomal circ0000337 contributes to DDP resistance in esophageal cancer by regulating Janus kinase 2 (JAK2) signaling via miR-377-3p (122), CRC-derived exosomal circ-0006174 (123) and circ-0000338 (124) contribute to doxorubicin and 5-fluorouracil chemoresistance. Similarly, exosomal circ-0032821 (125) has been reported to increase oxaliplatin (OXA) resistance in GC cells, and exosomal plasmacytoma variant translocation 1 circRNA (circPVT1) was shown to be significantly associated with increased DDP resistance in GC (126). In summary, as tumor resistance emerges with tumor treatment, establishing exosomal circRNA markers that can predict the onset and changes of drug resistance will be important for the precision diagnosis and treatment of tumors.

## 6 Conclusion and perspectives

In the TME, HCC cells undergo a complex immune escape process in which tumor and immune cells play major roles, with exosomal circRNAs acting as indispensable mediators between them. Immunotherapy is an emerging approach for the treatment of tumors that protects immune cells and enhances the body’s immunity by blocking various immunosuppressive mechanisms in the TME. Immune checkpoint inhibitors that are widely used clinically include PD-1/PD-L1 and CTLA-4. This review confirmed that exosomal circRNAs act on immune cells to help HCC cells achieve immune escape, and they play an important role in regulating tumor cell proliferation, metastasis, and drug resistance. More importantly, circRNAs have highly conserved profiles and tissue/development-specific expression patterns that can be enriched in exosomes and detected in bodily fluids. Thus, exosomal circRNAs could be used as novel immune checkpoint inhibitors or therapeutic targets. However, only a few circRNAs identified to date have clear functional and clinical applications. Compared to that of mRNAs and miRNAs, the present understanding of exosomal circRNAs remains limited. With continuous improvements in detection methods, experimental methodologies, and bioinformatics algorithms, it is believed that exosomal circRNAs will play a key role in tumor diagnosis and treatment, with future studies providing better guidance for clinical treatment and the realization of their use in precision medicine.

## Author contributions

TX and SL provided direction and guidance for this manuscript. HZX wrote the whole manuscript. All authors contributed to the article and approved the submitted version.
